# The Essential Role of Rac1 Glucosylation in *Clostridioides difficile* Toxin B-Induced Arrest of G1-S Transition

**DOI:** 10.3389/fmicb.2022.846215

**Published:** 2022-03-07

**Authors:** Lara Petersen, Svenja Stroh, Dennis Schöttelndreier, Guntram A. Grassl, Klemens Rottner, Cord Brakebusch, Jörg Fahrer, Harald Genth

**Affiliations:** ^1^Institute for Toxicology, Hannover Medical School, Hannover, Germany; ^2^Department of Toxicology, University Medical Center Mainz, Mainz, Germany; ^3^Institute of Medical Microbiology and Hospital Epidemiology and DZIF partner site Hannover, Hannover Medical School, Hannover, Germany; ^4^Division of Molecular Cell Biology, Zoological Institute, Technische Universität Braunschweig, Braunschweig, Germany; ^5^Department of Cell Biology, Helmholtz Centre for Infection Research, Braunschweig, Germany; ^6^Biotech Research and Innovation Centre (BRIC), University of Copenhagen, Copenhagen, Denmark; ^7^Rudolf-Buchheim-Institute of Pharmacology, Justus-Liebig-University Giessen, Giessen, Germany

**Keywords:** cyclin D, cell cycle, p21-activated kinase, human intestinal organoids, large clostridial glucosylating toxins, colonic epithelial renewal, human colonic epithelial cells

## Abstract

*Clostridioides difficile* infection (CDI) in humans causes pseudomembranous colitis (PMC), which is a severe pathology characterized by a loss of epithelial barrier function and massive colonic inflammation. PMC has been attributed to the action of two large protein toxins, Toxin A (TcdA) and Toxin B (TcdB). TcdA and TcdB mono-O-glucosylate and thereby inactivate a broad spectrum of Rho GTPases and (in the case of TcdA) also some Ras GTPases. Rho/Ras GTPases promote G1-S transition through the activation of components of the ERK, AKT, and WNT signaling pathways. With regard to CDI pathology, TcdB is regarded of being capable of inhibiting colonic stem cell proliferation and colonic regeneration, which is likely causative for PMC. In particular, it is still unclear, the glucosylation of which substrate Rho-GTPase is critical for TcdB-induced arrest of G1-S transition. Exploiting SV40-immortalized mouse embryonic fibroblasts (MEFs) with deleted Rho subtype GTPases, evidence is provided that Rac1 (not Cdc42) positively regulates Cyclin D1, an essential factor of G1-S transition. TcdB-catalyzed Rac1 glucosylation results in Cyclin D1 suppression and arrested G1-S transition in MEFs and in human colonic epithelial cells (HCEC), Remarkably, Rac1^−/−^ MEFs are insensitive to TcdB-induced arrest of G1-S transition, suggesting that TcdB arrests G1-S transition in a Rac1 glucosylation-dependent manner. Human intestinal organoids (HIOs) specifically expressed Cyclin D1 (neither Cyclin D2 nor Cyclin D3), which expression was suppressed upon TcdB treatment. In sum, Cyclin D1 expression in colonic cells seems to be regulated by Rho GTPases (most likely Rac1) and in turn seems to be susceptible to TcdB-induced suppression. With regard to PMC, toxin-catalyzed Rac1 glucosylation and subsequent G1-S arrest of colonic stem cells seems to be causative for decreased repair capacity of the colonic epithelium and delayed epithelial renewal.

## Introduction

The *Clostridioides difficile* Toxin B (TcdB, 270 kDa) [together with Toxin A (TcdA, 307 kDa)] is causative for the pathology of *C. difficile* infection (CDI), ranging from mild diarrhea to pseudomembranous colitis (PMC; Smits et al., 2016). PMC is characterized by a loss of epithelial barrier function and massive colonic inflammation. TcdA and TcdB are large single-chain protein toxins with a multi-domain organization, allowing self-mediated entry of the N-terminal glucosyltransferase domain into mammalian target cells by receptor-mediated endocytosis (Aktories et al., 2017; Kordus et al., 2021; Orrell and Melnyk, 2021). Upon internalization into the cytosol, the glucosyltransferase domain catalyzes divalent metal ion-dependent mono-O-glucosylation and thereby inactivation of small GTPases of Rho and Ras families (Genth et al., 2014, 2016, 2018). While TcdB specifically glucosylates GTPases of the Rho, Rac, and Cdc42 subfamilies, the related TcdA exhibits a broader substrate profile, as it glucosylates Ras subtype GTPases in addition to Rho/Rac/Cdc42 subtype GTPases (Genth et al., 2018). Ras and Rho GTPases are the key regulators of cytoskeletal dynamics, of gene expression, of cell cycle progression, and of cell death/survival. Upon treatment of cultured cells with TcdB, glucosylation of Rho GTPases results in actin depolymerization (Mitchell et al., 1987; May et al., 2013) and cell death including upregulation of the cell death-regulating GTPase RhoB (Huelsenbeck et al., 2007; Matarrese et al., 2007; Farrow et al., 2013; Wohlan et al., 2014).

In the gastrointestinal tract, a single layer of epithelial cells and the mucus layer separate the body from adverse factors in the gut environment. The gastrointestinal tract, however, is vulnerable to damage induced by adverse factors including bacterial toxins (such as TcdA and TcdB), metabolites (such as cytotoxic bile acids), dietary antigens and carcinogens, and oxidative stress. Likely because of this vulnerability, the intestinal epithelium undergoes rapid self-renewal with a renewal cycle of 4–5 days. This renewal depends on stem cells that feed the compartment of rapidly cycling transit-amplifying (TA) cells, which divide 4–5 times prior to their differentiation upon crossing the crypt villus junction. The proliferative activity and the acquisition of particular cell fates are coordinated by a small number of conserved signaling pathways, including the Wnt/beta-catenin and the Notch signaling pathways (de Lau et al., 2014; Reynolds et al., 2014).

Cell cycle progression requires sequential expression of cyclins, which complexes specific cyclin-dependent kinases (Cdks). Cyclin D1 is one of the essential cyclins that (upon binding to Cdk4/6) regulate G1-S transition during normal cell-cycle progression. p21-activated kinase (PAK) promotes G1-S transition through activation of components of the ERK, AKT and Wnt signaling pathways, all of which regulate expression of Cyclin D isoforms (Radu et al., 2014). PAK1 and PAK2 are activated by Rho subfamily GTPases Rac1 and Cdc42 (Bokoch, 2003; Parri and Chiarugi, 2010).

TcdA and TcdB induce a G1-S arrest in a glucosylation-dependent manner, with concomitant glucosylation of RhoA, Rac1, and Cdc42 being regarded to be required for arrested G1-S transition (Lica et al., 2011; D’Auria et al., 2012; Wohlan et al., 2014; Fettucciari et al., 2017; He et al., 2017). This study sets out to identify those Rho GTPases regulating Cyclin D1 expression and G1-S transition. Exploiting Rac1 and Cdc42 knock-out fibroblasts, glucosylation of specifically Rac1 (not Cdc42) is shown to be required for TcdB-induced Cyclin D1 suppression and arrested G1-S transition. Furthermore, evidence on the inhibition of Rac/Cdc42-PAK signaling and subsequent Cyclin D1 suppression is provided in TcdB-treated human colonic epithelial cells (HCEC) and in human intestinal organoids (HIO).

## Materials and Methods

### Materials

Commercially obtained reagents: TO-PRO-3 (Life Technologies, Darmstadt, Germany); [^32^P]NAD; Staurosporine. Antibodies: RhoA (26C4; Santa Cruz); Rac1 (clone 102, Transduction Laboratories); Rac1 (clone 23A8, Millipore); beta-actin (AC-40, Sigma); Hsp90 (Sigma); pS144/141-PAK1/2 (EP656Y, Abcam); PAK2 (Cell Signaling); Cyclin D1 (Cell Signaling); Cdc42 (Transduction Laboratories); horseradish peroxidase conjugated secondary antibody, mouse (Rockland); horseradish peroxidase conjugated secondary antibody, rabbit (Rockland). Recombinant TcdB and TcdB-NxN were expressed in *Bacillus megaterium* and purified by Ni^2+^ affinity chromatography using Ni^2+^-IDA columns (Macherey-Nagel, Germany; Burger et al., 2003; Wohlan et al., 2014).

### Cell Culture

Human intestinal organoids were grown from surgically removed intestinal tissue as recently described (Schöttelndreier et al., 2018). Non-transformed human colonic epithelial cells (HCEC) were kindly provided by Dr. Jerry Shay (Department of Cell Biology, UT Southwestern Medical Center, Dallas, United States) in 2015. HCEC exhibit a cuboidal to spindle-shaped morphology and intact p53 signaling (Mimmler et al., 2016). HCEC were maintained as previously described and grown in a nitrogen incubator with reduced oxygen levels (Roig et al., 2010). SV40 LT-antigen-immortalized Rac1^−/−^ (clone 3) and Rac1^fl/fl^ (Steffen et al., 2013) and LT-antigen-immortalized Cdc42^−/−^ (clone 3-9-9) and Cdc42^fl/−^ (clone 3–9; Czuchra et al., 2005) mouse embryonic fibroblasts were cultivated under standard conditions in Dulbecco’s modified essential medium supplemented with 10% fetal calf serum, 100 μM penicillin, 100 μM streptomycin, 1% non-essential amino acids (*NEAA*), and 1 mM sodium pyruvate at 37°C and 5% CO_2_. Cells were passaged upon 80% confluence. Doubling times of either cell line were estimated by dividing the natural logarithm of two by the exponent of growth.

### EdU Labeling of Replicating Cells and Confocal Microscopy

HCEC grown on coverslips were incubated with TcdB for 24 h or left untreated. As positive control, cells were starved in serum-free medium. Cells were then labeled with EdU for 1 h. After fixation and blocking, the samples were incubated with the Click-iT® EdU Imaging Kit (Life Technologies, Darmstadt, Germany) for 1 h in the dark. After a washing step, nuclei were counterstained with TO-PRO-3 (Life Technologies, Darmstadt, Germany) and samples mounted using Vectashield® (Vector Labs, Burlingame, CA, United States). Confocal microscopy was performed using a Zeiss Axio Observer.Z1 microscope equipped with a LSM710 laser-scanning unit (Zeiss, Oberkochen, Germany). Images were acquired in optical sections of 1 μm and processed with ImageJ (NIH, USA). The number of EdU-positive cells was quantified with ImageJ (50–100 cells/treatment) and data were evaluated by using the GraphPad Prism software.

### BrdU Labeling of Replicating Cells

The BrdU Cell Proliferation Assay (Merck Millipore, Darmstadt, Germany) was exploited for the analysis of G1-S transition. The assay was performed according to the manufacturers’ instructions. In brief, proliferating cells were pre-loaded with BrdU, followed by TcdB treatment for 24 h. Thereby, BrdU was incorporated into the DNA of proliferating cells. Cells were fixed, permeabilized, and the labeled DNA was denatured to enable binding of the anti-BrdU monoclonal antibody. A secondary, horseradish peroxidase-labeled antibody catalyzes the conversion of the chromogenic substrate tetra-methylbenzidine (TMB) from a colorless solution to a blue solution (or yellow after the addition of stopping reagent). The level of the colored reaction product was quantified by photometry using a scanning multiwell spectrophotometer.

### Western Blot Analysis

Cells were seeded into 3.5-cm-diameter dishes, treated with TcdB or mock for 24 h and washed with phosphate buffered saline (PBS; 4°C). For western blot analysis, cells were lysed with a buffer containing HEPES (50 mM), NaCl (150 mM), MgCl_2_ (5 mM), Laemmli sample buffer, PMSF (1 mM) and sodium vanadate pH 7.4. The suspension was sonified with 50% cycle for 5 s. Subsequently, the samples were incubated at 95°C for 10 min, centrifuged, and separated by SDS-PAGE (15% gels, 25 mA/gel). For Western Blotting, proteins were transferred onto nitrocellulose membranes, which were blocked with 5% (w/v) non-fat dried milk for 60 min and incubated with respective primary antibody overnight at 4°C. Following treatment of the membrane with the second antibody for 60 min, proteins were detected using ECL Femto (Pierce).

### Sequential [^32^P]ADP Ribosylation

For sequential [^32^P]ADP ribosylation, cells were lysed in a buffer containing 150 mM NaCl, 50 nM Tris (pH 7.2), 5 mM MgCl_2_, 1 mM PMSF and NP40 (1%). After sonification, the soluble fraction was prepared by centrifugation. The lysates were incubated with *C. botulinum* exoenzyme C3 in the presence of 0.3 μM [^32^P]NAD and 3 μM NAD for 30 min at 37°C. The reaction was stopped by addition of Laemmli sample buffer. The samples were incubated at 95°C for 10 min, separated by SDS-PAGE and analysed using a PhosphoImager.

## Results

### Cyclin D1 Suppression in TcdB-Treated Human Intestinal Organoids and Human Colonic Epithelial Cells

**Figure 1:**
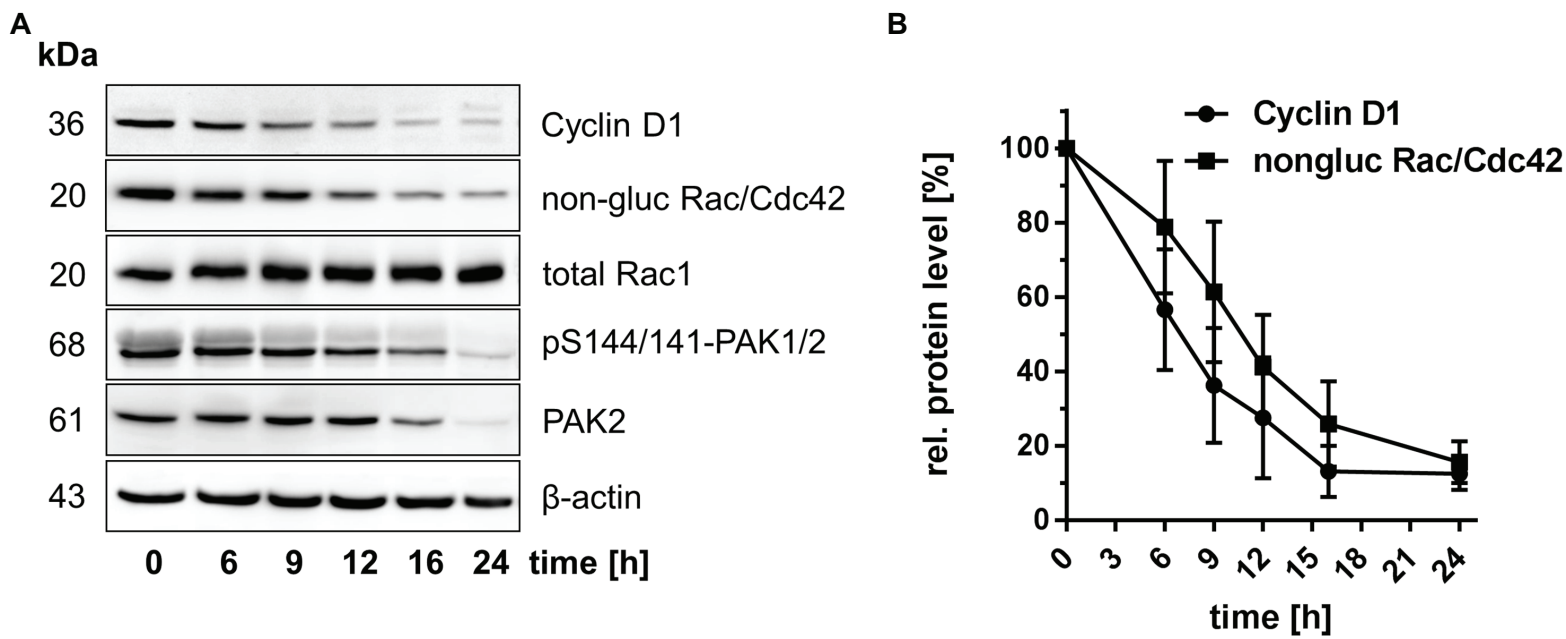
Suppression of Cyclin D1 in TcdB-treated human intestinal organoids (HIOs). **(A)** HIOs were treated with TcdB (30 ng/ml) for the indicated times. The relative cellular concentrations of the indicated proteins were determined using Western blot analysis. Representative Western blots are presented. **(B)** Quantifications of signals were performed using Kodak software. Signal intensities obtained from immunoblots blots (n = 3) were quantified and normalized to the signal of beta-actin. The concentration of the indicated proteins in non-treated cells was set 100. Values are given as mean ± SD of three independent experiments.

Human intestinal organoids (HIOs) were exploited as experimental model for studying the effect of TcdB on the expression of the G1-phase Cyclins Cyclin D1, Cyclin D2, and Cyclin D3. HIOs specifically expressed Cyclin D1 but neither Cyclin D2 nor Cyclin D3 (Supplementary Figure S1). In contrast, SV40-immortalized Rac1^fl/fl^ mouse embryonic fibroblasts (MEFs) that expressed Cyclin D1, Cyclin D2, and Cyclin D3 served as a positive control (Supplementary Figure S1). Treatment of HIOs with TcdB caused a time-dependent suppression of Cyclin D1 (Figures 1A,B). TcdB-catalyzed glucosylation of Rac/Cdc42 was evaluated by immunoblot analysis using Rac1 antibody (mAb102) that detects Rac/Cdc42 (Genth et al., 2006; Brandes et al., 2012). Once Rac/Cdc42 is glucosylated by TcdB, this Rac1 antibody (mAb102) does not detect its epitope, resulting in signal loss (Genth et al., 2006; Brandes et al., 2012). Decreased detection of Rac/Cdc42 by this antibody thus reflects TcdB-catalyzed glucosylation of Rac/Cdc42. TcdB time-dependently glucosylated Rac/Cdc42 (Figures 1A,B), as evaluated in terms of the loss of the detection of Rac/Cdc42 by immunoblot analysis using Rac1 antibody (mAb102). The total cellular level of Rac1 was not changed upon TcdB treatment as assessed using the Rac1 antibody (mAb23A8; Figure 1A), confirming that decreasing detection of Rac/Cdc42 by the Rac1 antibody (mAb102) was due to glucosylation and not due to degradation. Rac/Cdc42 from HIOs are thus susceptible to TcdB-catalyzed glucosylation, consistent with a former report (Zhu et al., 2019). PAK1/2, the upstream regulator of Cyclin D, is activated by Rac/Cdc42 (Balasenthil et al., 2004; Thullberg et al., 2007). In turn, TcdB-induced Rac/Cdc42 glucosylation resulted in a time-dependent decrease of phospho-Ser-144-PAK1 and phospho-Ser-141-PAK2 (pS144/141-PAK1/2; Figure 1A), with PAK1/2 dephosphorylation being indicative of PAK1/2 deactivation, consistent with previous observations (May et al., 2014). The total levels of PAK2 also decreased (Figure 1A), showing that TcdB-induced pS144/141-PAK1/2 deactivation was based on both PAK dephosphorylation and PAK degradation. In TcdB-treated HIOs, inhibition of Rac/Cdc42-PAK signaling by TcdB, thus coincided with suppression of Cyclin D1, with the kinetics of Cyclin D1 suppression being almost comparable to that of Rac/Cdc42 glucosylation (Figures 1A,B). The observation that TcdB-induced inhibition of Rac/Cdc42-PAK signaling results in suppression of Cyclin D1 reinforces the paradigm that expression of Cyclin D1 is regulated by a Rac/Cdc42-PAK-dependent signaling pathway (Joyce et al., 1999; Klein et al., 2007).

**Figure 2:**
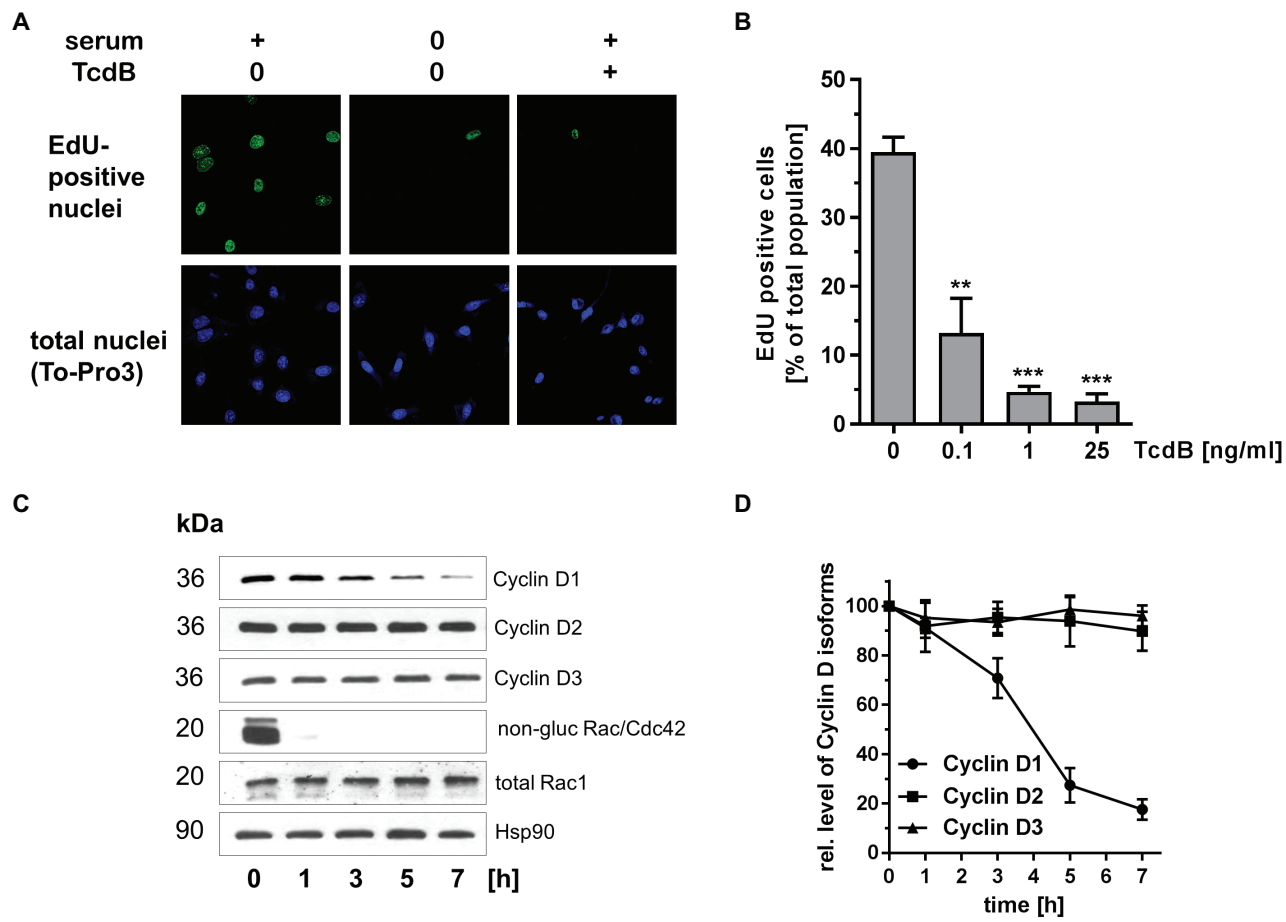
Effects of TcdB in human colonic epithelial cells (HCEC). **(A)** HCEC were treated with TcdB (25 ng/ml) or maintained in serum-starved medium as positive control for 24 h. Actively replicating S-phase cells were labeled with EdU and visualized by confocal microscopy. Upon fixation of cells, nuclei were counterstained with TO-PRO-3. **(B)** HCEC were treated with the indicated concentrations of TcdB for 24 h. Actively replicating S-phase cells were labeled with EdU and visualized by confocal microscopy. The number of EdU-positive per TO-PRO-3-positive nuclei was determined. Signals from EdU-positive cells were quantitatively evaluated using ImageJ software (*n* = 3). ***p < 0.001, **p < 0.01. **(C)** HCEC were treated with TcdB (25 ng/ml) for the indicated time. The relative cellular concentrations of the indicated proteins were determined using Western blot analysis. Representative Western blots are presented. **(D)** Signal intensities obtained from the Western blots (*n* = 3) were quantified and normalized to the signal of Hsp90. The concentration of the indicated proteins in non-treated cells was set 100. Values are given as mean ± SD of three independent experiments.

Next, the TcdB effects on G1-S transition were analyzed in human colonic epithelial cells (HCEC) isogenetically transformed with Cdk4 and human telomerase. HCEC are a frequently exploited model to recapitulate colorectal cancer initiation and progression (Roig et al., 2010; Zhang et al., 2015; Mimmler et al., 2016). Treatment of HCEC with TcdB resulted in cell rounding with the formation of bipolar retraction fibers (Supplementary Figure S2), showing that HCEC are sensitive to TcdB. S-phase entry of HCEC was analyzed in terms of EdU incorporation. About 40% of a serum-maintained non-synchronously proliferating population of HCEC was EdU-positive (Figures 2A,B), reflecting those cells passing G1-S transition and S-phase within 24 h. Serum depletion resulted in an almost complete loss of EdU-positive cells, showing that in the absence of growth factors HCEC were arrested at the G1-S boundary (Figure 2A). TcdB concentration-dependently reduced the number of EdU-positive cells, indicating blocked G1-S transition (Figures 2A,B). HCEC expressed the G1-phase Cyclins Cyclin D1, Cyclin D2, and Cyclin D3 (Figure 2C). Interestingly, specifically the expression of Cyclin D1 (neither of Cyclin D2 nor Cyclin D3) was suppressed in TcdB-treated HCEC (Figures 2C,D). At the chosen relatively high TcdB concentration of 25 ng/ml, TcdB completely glucosylated Rac/Cdc42 within 1 h of TcdB treatment (Figure 2C), as evaluated in terms of the loss of the detection of Rac/Cdc42 Western by immunoblot analysis using Rac1 antibody (mAb102). The cellular level of Rac1 was not changed upon TcdB treatment as assessed using the Rac1 antibody (mAb23A8; Figure 2C), confirming that decreasing detection of Rac/Cdc42 by the Rac1 antibody (mAb102) was due to glucosylation and not due to degradation. Furthermore, prolonged treatment of HCEC with TcdB for 24 h did not result in Cyclin D2 and Cyclin D3 suppression (Supplementary Figure S3). In proliferating HCEC, Rac/Cdc42 glucosylation appeared earlier as compared to Cyclin D1 degradation (Figures 2C,D). The latter observation is consistent with the current paradigm stating that a Rac1-dependent pathways leads to transcriptional activation of cyclin D1 (Joyce et al., 1999; Klein et al., 2007). In turn, Rac1 glucosylation most likely blocks transcriptional cyclin D1 activation, resulting in degradation of pre-formed Cyclin D1. TcdB-induced Rac/Cdc42 glucosylation thus coincided with suppression of specifically Cyclin D1 and arrested G1-S transition in HCEC.

### Suppression of Cyclin D1 and Cyclin D2 Correlates With Increased Doubling Time of Rac1^−/−^ MEFs

**Figure 3:**
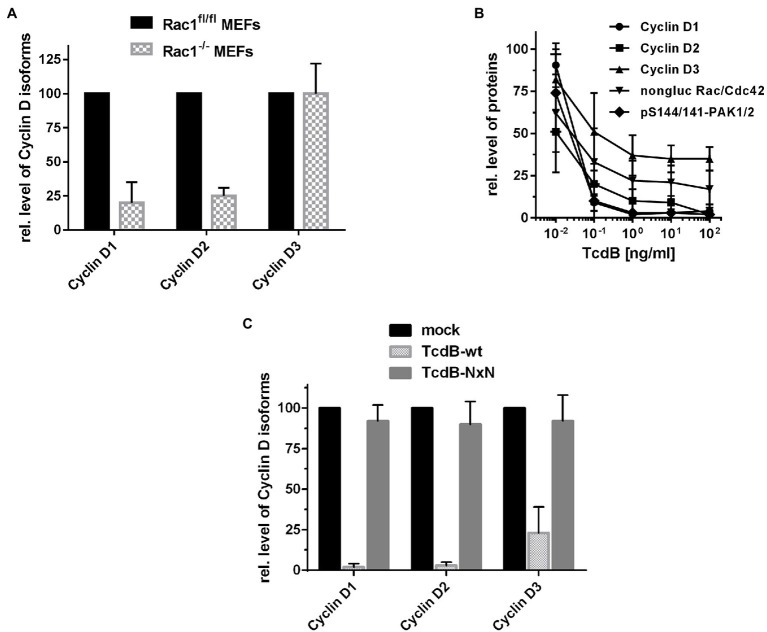
Expression of Cyclin D isoforms upon Rac1 deletion and TcdB treatment. **(A)** Lysates from proliferating Rac1^fl/fl^ and Rac1^−/−^ MEFs were analyzed for the cellular concentrations of the Cyclin D isoforms using Western blot analysis. **(B)** Proliferating Rac1^fl/fl^ MEFs were treated with the indicated concentrations of TcdB for 24 h. **(C)** Proliferating Rac1^fl/fl^ MEFs were treated with TcdB-wt and TcdB-NxN (1 ng/ml each) or mock for 24 h. The relative cellular concentrations of the indicated proteins were determined using Western blot analysis. Signal intensities obtained from Western blots (*n* = 3) were quantified and normalized to the signal of beta-actin. The concentration of the indicated proteins in non-treated cells was set 100. Values are given as mean ± SD of three independent experiments.

To check if the inhibition of either Rac1 or Cdc42 is sufficient for suppression of Cyclin D1, SV40-immortalized mouse embryonic fibroblasts (MEFs) with a genetic deletion of either Rac1 or Cdc42 were exploited (Czuchra et al., 2005; Steffen et al., 2013). SV40-immortalized Rac1^fl/fl^ MEFs were rapidly proliferating cells with a doubling time of about 13 h (Supplementary Figure S4A), as estimated by dividing the natural logarithm of two by the exponent of growth. In contrast, Rac1^−/−^ MEFs exhibited a doubling time of about 24 h (Supplementary Figure S4A), indicating delayed cell cycle progression. Delayed cell cycle progression of Rac1^−/−^ MEFs coincided with suppressed expression of the G1 Cyclins Cyclin D1 and Cyclin D2 in non-treated Rac1^−/−^ MEFs (Figure 3A; Supplementary Figure S5A). In contrast to Cyclin D1 and Cyclin D2, Cyclin D3 was expressed in non-treated Rac1^−/−^ MEFs to a level comparable to that in Rac1^fl/fl^ (Figure 3A; Supplementary Figure S5A). The presence of Cyclin D3 might ensure G1-S transition and (delayed) proliferation of non-treated Rac1^−/−^ MEFs, based on the idea that Cyclin D3 compensates for inactivation or loss of Cyclin D1 (Radulovich et al., 2010; Zhang et al., 2011). In contrast to Rac1^fl/fl^ and Rac1^−/−^ MEFs, SV40-immortalized Cdc42^fl/−^ and Cdc42^−/−^ MEFs proliferated with almost comparable doubling times of about 14 h and 16 h, respectively (Supplementary Figure S4B). The level of Cyclin D1 appeared to be increased in non-treated Cdc42^−/−^ MEFs as compared to non-treated Cdc42^fl/−^ MEFs (Supplementary Figure S6) for reasons that remain to be unclear. Notwithstanding this, our observations argued for a major function of Rac1 (not Cdc42) in positively regulating Cyclin D1 expression and G1-S transition of SV40-immortalized fibroblasts.

### Lacking Susceptibility of Rac1^−/−^ MEFs to TcdB-Induced Arrest of G1-S Transition

The observation that Cyclin D1 and Cyclin D2 are suppressed in Rac1^−/−^ MEFs (Figure 3) led to the hypothesis that TcdB-catalyzed Rac1 glucosylation (i.e., Rac1 inactivation) is causative for Cyclin D1 and Cyclin D2 suppression. Upon TcdB treatment for 24 h, the levels of Cyclin D1 and Cyclin D2 TcdB concentration-dependently decreased in Rac1^fl/fl^ MEFs, with a TcdB concentration of 1 ng/ml being sufficient for almost complete Cyclin D1 and Cyclin D2 suppression (Figure 3B; Supplementary Figure S5A). In contrast, Cyclin D3 expression was reduced to some extent in TcdB-treated Rac1^fl/fl^ MEFs but not completely suppressed even at high TcdB concentrations (Figure 3B; Supplementary Figure S5A).

**Figure 4:**
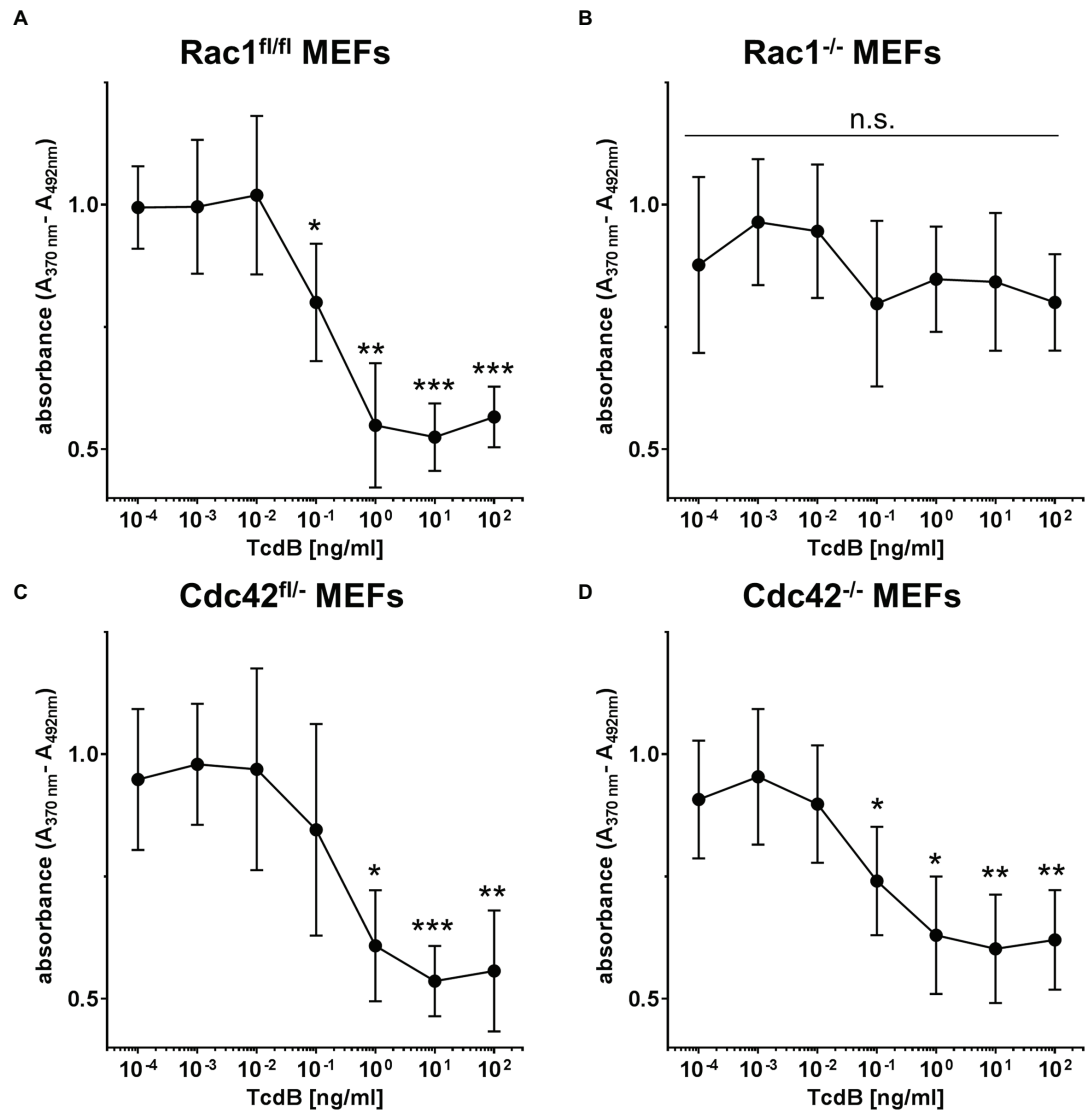
TcdB effects on G1-S transition. Proliferating Rac1^fl/fl^ MEFs **(A)**, Rac1^−/−^ MEFs **(B)**, Cdc42^fl/−^ MEFs **(C)**, and Cdc42^−/−^ MEFs **(D)** were labeled with BrdU (10 μM) and treated with the indicated concentrations of TcdB for 24 h. DNA *de novo* synthesis is determined using a peroxidase-conjugated anti-BrdU antibody. Values are given as mean ± SD of three independent experiments. ***p < 0.001, **p < 0.01, *p < 0.05.

To check if TcdB-induced suppression of Cyclin D1 and Cyclin D2 coincides with arrested G1-S transition, BrdU incorporation (indicative of S-phase entry) into non-synchronously proliferating cells was investigated. TcdB concentration-dependently reduced BrdU incorporation into non-synchronously proliferating Rac1^fl/fl^ MEFs (Figure 4A). Remarkably, BrdU incorporation into non-synchronously proliferating Rac1^−/−^ MEFs was not responsive to TcdB treatment (Figure 4B). These observations strongly suggest that TcdB inhibits G1-S transition in a Rac1 glucosylation-dependent manner. TcdB concentration-dependently reduced BrdU incorporation into both Cdc42^fl/−^ and Cdc42^−/−^ MEFs (Figures 4C,D). In contrast to Rac1^−/−^ MEFs, Cdc42^−/−^ MEFs did not lack responsiveness to TcdB, most likely excluding a role of Cdc42 glucosylation in TcdB-induced G1-S arrest. TcdB-induced inhibition of G1-S transition depended on Rac1 glucosylation and coincided with suppression of Cyclin D1 and Cyclin D2.

TcdB exhibited comparable potency in Rac1^fl/fl^ and Rac1^−/−^ MEFs, as TcdB concentration-dependent RhoA glucosylation was comparable in Rac1^fl/fl^ MEFs and Rac1^−/−^ MEFs, with a TcdB concentration of 1 ng/ml being sufficient for almost complete RhoA glucosylation (Supplementary Figures S5A,B). RhoA/B/C glucosylation was thereby tracked in terms of sequential [^32^P]ADP-ribosylation exploiting *C. botulinum* exoenzyme C3. Once RhoA/B/C is glucosylated by TcdB at Thr-37, sequential ADP-ribosylation of RhoA/B/C is blocked, resulting in a loss of [^32^P]ADP-ribosylation (Just et al., 1994). Decreasing [^32^P]ADP-ribosylation of RhoA/B/C thus reflects TcdB-catalyzed glucosylation of RhoA/B/C (Supplementary Figures S5A,B). Moreover, Cdc42 degradation, PAK de-activation, and RhoA/B/C glucosylation exhibited comparable kinetics in TcdB-treated Rac1^fl/fl^ and Rac1^−/−^ MEFs (Supplementary Figures S5A,B). These observations provide evidence on comparable TcdB potency in Rac1^fl/fl^ and Rac1^−/−^ MEFs. TcdB also exhibited comparable potency in Cdc42^fl/−^ and Cdc42^−/−^ MEFs, as RhoA glucosylation, RhoA degradation, PAK de-activation, and PAK degradation exhibited comparable kinetics in Cdc42^fl/−^ and Cdc42^−/−^ MEFs (Supplementary Figure S6).

In Rac1^−/−^ MEFs, low levels of Cyclin D1 were still expressed, with this residual Cyclin D1 expression being sensitive to TcdB treatment (Supplementary Figure S5A; Cyclin D1*). The residual Cyclin D1 expression in Rac1^−/−^ MEFs seems to be regulated by a TcdB substrate GTPase distinct from Rac1. Appropriate candidates are RhoA and the Cdc42 subfamily GTPase TC10, both of which are glucosylated by TcdB and are shown to regulate Cyclin D1 expression (Murphy et al., 2001; Croft and Olson, 2006; Genth et al., 2014).

The low expression of Cyclin D1 and Cyclin D2 does not seem to regulate G1-S progression in Rac1^−/−^ MEFs, as Rac1^−/−^ MEFs progressed through the G1-S transition at those TcdB concentrations being sufficient for complete Cyclin D1 suppression (Supplementary Figure S5A; Figure 4B). Instead, G1-S transition in Rac1^−/−^ MEFs might be promoted by Cyclin D3, the expression of which is only reduced to some extent upon TcdB treatment (Figure 3B; Supplementary Figure S5A).

TcdB harbors two adjacent aspartates at acid position 286 and 288, which mediate divalent metal ion-dependent coordination of the glucose donor UDP-glucose. Upon exchange of D286 and D286 into asparagins (TcdB-NxN), the glucosyltransferase activity is deleted (Wohlan et al., 2014). To confirm that TcdB-induced suppression of the Cyclin D isoforms depends on the glucosyltransferase activity of TcdB, Rac1^fl/fl^ MEFs were treated TcdB with as a positive control. TcdB caused rounding of Rac1^fl/fl^ MEFs (Supplementary Figure S7A), suppression of the Cyclin D isoforms (Figure 3C; Supplementary Figure S7B), Rac/Cdc42 glucosylation (Supplementary Figure S7B), and PAK deactivation (Supplementary Figure S7B). In contrast, TcdB-NxN-treated Rac1^fl/fl^ MEFs exhibited expression of the Cyclin D isoforms and active Rac/Cdc42-PAK signaling, as neither Rac/Cdc42 glucosylation nor Cdc42 degradation nor PAK deactivation were observed (Figure 3C; Supplementary Figure S7B). These findings confirmed that TcdB-induced suppression of Cyclin D isoforms depended on the glucosyltransferase activity of TcdB.

### Susceptibility of Rac1^fl/fl^ and Rac1^−/−^ MEFs to Staurosporine-Induced Arrest of G1-S Transition

To show that G1-S transition in Rac1^−/−^ MEFs is in principle susceptible to inhibition, staurosporine (STS), a broad-spectrum inhibitor of protein kinases, was applied. STS concentration-dependently reduced BrdU incorporation into both non-synchronously proliferating Rac1^fl/fl^ MEFs and Rac1^−/−^ MEFs (Supplementary Figure S8A). STS concentration-dependently induced decreasing levels of pT202/Y204-p44-MAPK(ERK1) and pT183/Y185-p42-MAPK(ERK2) in both Rac1^fl/fl^ and Rac1^−/−^ MEFs (Supplementary Figure S8B), confirming that STS acts as a kinase inhibitor. The total levels of ERK1/2 concentration-dependently decreased (Supplementary Figure S8B), showing that STS-induced ERK1/2 de-activation is based on both de-phosphorylation and degradation. ERK1/2 is an up-stream regulator of Cyclin D1 expression and G1-S transition (Baker et al., 2014; Radu et al., 2014). Furthermore, STS concentration-dependently induced Cyclin D1 suppression in Rac1^fl/fl^ MEFs (Supplementary Figure S8B). In STS-treated Rac1^fl/fl^ MEFs, Cyclin D1 suppression thus coincided with arrested G1-S transition. The responsiveness of G1-S transition in Rac1^−/−^ MEFs to STS proved that Rac1^−/−^ MEFs were in principle susceptible to a G1-S arrest.

## Discussion

The Rac1/Cdc42-PAK pathway promotes G1-S transition through the activation of components of the ERK, AKT, and Wnt signaling pathways, all of which regulate expression of Cyclin D isoforms (Radu et al., 2014). In this study, MEFs with genetic deletion of either Rac1 or Cdc42 were exploited to provide evidence that the genetic deletion of Rac1 (not Cdc42) results in suppression of Cyclin D1 and Cyclin D2 (Figure 3). Consistently, suppression of Cyclin D1 and D2 coincides with an increased doubling time of Rac1^−/−^ MEFs (Supplementary Figure S4). As delayed G2-M transition hardly contributes to the increased doubling time of Rac1^−/−^ MEFs, delayed cell cycle progression of Rac1^−/−^ MEFs can be associated with delayed G1-S transition (May et al., 2014). Rac1 (not Cdc42) seems thus to be the upstream regulator of Cyclin D1 and Cyclin D2 and of G1-S transition in murine fibroblasts. Consistently, inactivation of Rac1 by TcdB-catalyzed glucosylation results in suppression of Cyclin D1 and Cyclin D2 and (to some extent) Cyclin D3 in MEFs (Figure 3B; Supplementary Figure S5A). These observations are consistent with former findings providing evidence on reduced expression of the cyclin d1 gene upon TcdB treatment (D’Auria et al., 2012). Furthermore, the related TcdA has been shown to suppress Cyclin D1 expression (Bezerra Lima et al., 2014). Interestingly, TcdA-induced inhibition of the Wnt/β-Catenin Pathway is shown to be preserved upon expression of non-glucosylatable Rac1-Q61L (Martins et al., 2020). In sum, Rac1 seems to be the critical upstream regulator of G1 phase Cyclins on the level of Rho GTPases, which glucosylation by either TcdA or TcdB results in Cyclin D1 suppression.

TcdB-induced suppression of Cyclin D1 and Cyclin D2 coincides with a G1-S arrest in MEFs (Figure 4), as evidenced in terms of reduced BrdU incorporation. These observations nicely complement former observations on TcdB-induced G1-S arrest, as analyzed in terms of a loss of the S-phase population and an increased 2 N population on FACS analysis of propidium iodide-stained cells (Nottrott et al., 2007; Lica et al., 2011; D’Auria et al., 2012; Wohlan et al., 2014). The observations that Rac1^−/−^ MEFs (not Cdc42^−/−^ MEFs) are insensitive to TcdB-induced arrest of G1-S transition (Figure 4) suggest that TcdB arrested G1-S transition in a Rac1 glucosylation-dependent manner. In Rac1^−/−^ MEFs, other Rho subfamily GTPases - dependently or independently of the “invisible hand” of Rho GDI-1-might compensate for the loss of Rac1. In TcdB-treated Rac1^−/−^ MEFs, such compensation can most likely be excluded, as TcdB completely inactivates the Rho, Rac, and Cdc42 subfamily GTPases (Boulter et al., 2010; Genth et al., 2018).

In human colonic epithelial cells (HCEC), expression of specifically Cyclin D1 (not Cyclin D2 or Cyclin D3) seems to depend on Rho subtype GTPases (most likely Rac1), as Cyclin D1 expression (not expression of Cyclin D2 and Cyclin D3) is sensitive to TcdB treatment (Figure 2). Comparable to MEFs, Cyclin D1 suppression coincides with arrested G1-S transition (Figure 2). In HCEC, TcdB-induced suppression of Cyclin D1 (not Cyclin D2 or D3) seems to be causative for the G1-S arrest.

**Figure 5:**
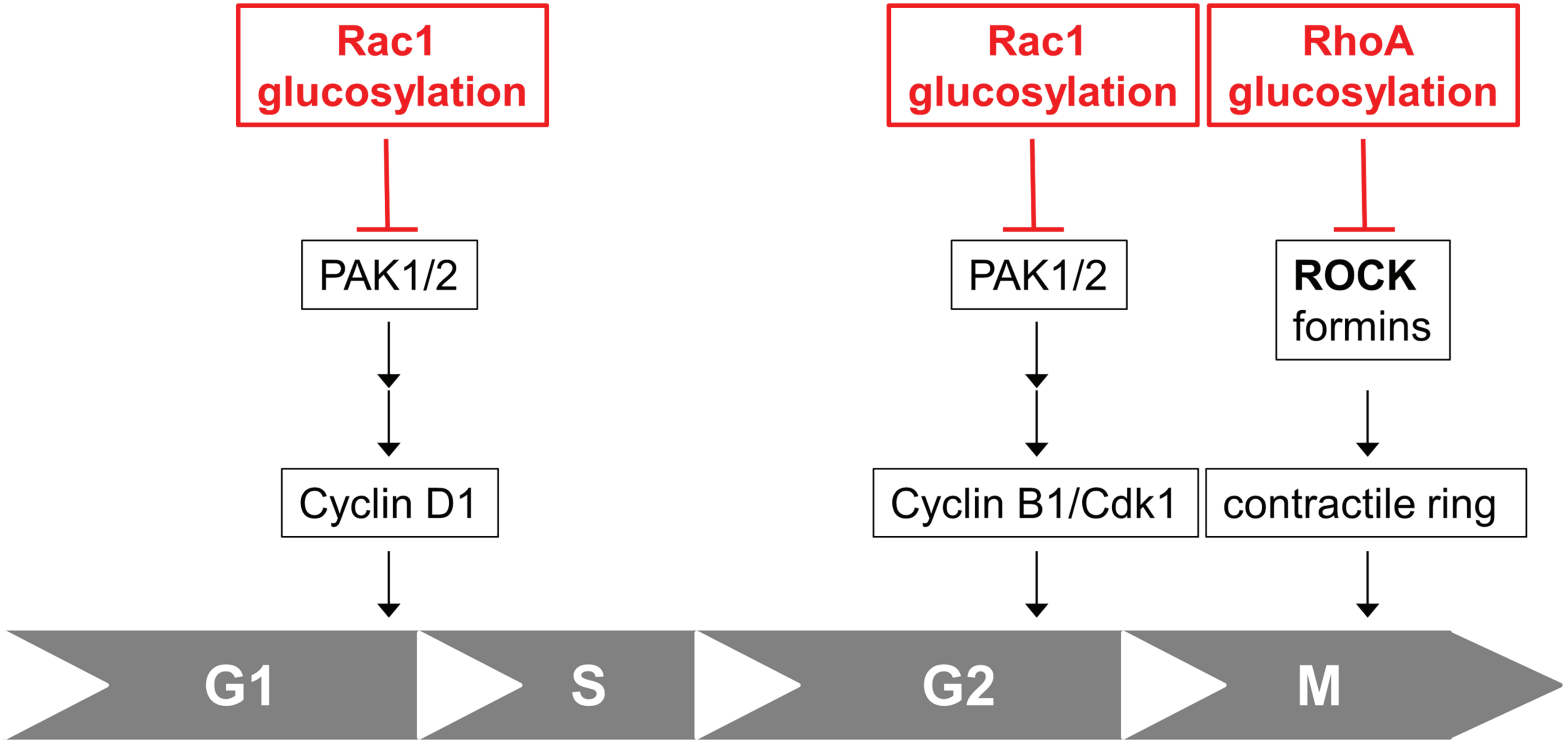
Cell cycle effects of TcdB. Rac1 glucosylation by TcdB results in inhibition of Rac1/PAK-dependent pathways regulating expression of Cyclin D1 and subsequent arrested G1-S transition. Rac1 glucosylation by TcdB also results in inhibition of Rac1/PAK-dependent pathways leading to Cyclin B/Cdk1 complex and subsequent delayed G2-M transition. RhoA glucosylation by TcdB blocks formation of the contractile actin-myosin ring (CAR) in cytokinesis, resulting in arrested cell division in M-phase.

Together with former studies on the cell cycle effect of TcdB, TcdB blocks cell proliferation by inhibition of several Rac1-/RhoA-dependent pathways regulating cell cycle progression: (1) TcdB-catalyzed Rac1 glucosylation results in delayed G2-M transition (Ando et al., 2007; May et al., 2014); (2) TcdB-catalysed RhoA glucosylation prevents contractile ring formation in cytokinesis, resulting in inhibited cell division (Huelsenbeck et al., 2009; Lica et al., 2011); (3) TcdB-induced G1-S arrest depends on Rac1 glucosylation (this study; Figure 5).

Several lines of evidence have highlighted Rac1 as a driver of colonic stem cell proliferation and colonic regeneration (Myant et al., 2013; McKernan and Egan, 2015). Most recently, TcdB (rather than TcdA) has been identified as the driver of colonic stem cell damage in CDI (Mileto et al., 2020). The observations of this study nicely complement the current model: Cyclin D1 seems to be the only G1 phase cyclin expressed in the colon, as deduced from our observations from HIOs (Supplementary Figure S1). TcdB-induced Rac1 glucosylation and PAK deactivation and subsequent suppression of Cyclin D1 and arrested G1-S transition seem to be causative for colonic damage, including a diminished LGR5+ stem cell compartment, and a damaged stem cell population. In consequence, the repair capacity of the colonic epithelium must be regarded to be decreased and the epithelial renewal to be delayed (Barker, 2014; Mileto et al., 2020).

## Data Availability Statement

The original contributions presented in the study are included in the article/Supplementary Material, and further inquiries can be directed to the corresponding author.

## Author Contributions

HG, JF, and GG conceived the study. LP, SS, and DS performed the experiments. KR and CB supplied reagents. LP, HG, JF, GG, and DS analyzed the data and wrote the manuscript. All authors contributed to the article and approved the submitted version.

## Funding

This work was funded by the Federal State of Lower Saxony, Niedersächsisches Vorab (VWZN3215/ZN3266).

## Conflict of Interest

The authors declare that the research was conducted in the absence of any commercial or financial relationships that could be construed as a potential conflict of interest.

## Publisher’s Note

All claims expressed in this article are solely those of the authors and do not necessarily represent those of their affiliated organizations, or those of the publisher, the editors and the reviewers. Any product that may be evaluated in this article, or claim that may be made by its manufacturer, is not guaranteed or endorsed by the publisher.
