# Supplementary material for: The Essential Role of Rac1 Glucosylation in Clostridioides difficile Toxin B-Induced Arrest of G1-S Transition
Source: Front Microbiol. 2022 Mar 7;13:846215. doi: 10.3389/fmicb.2022.846215 (PMC8937036; doi:10.3389/fmicb.2022.846215)

Fig. S1

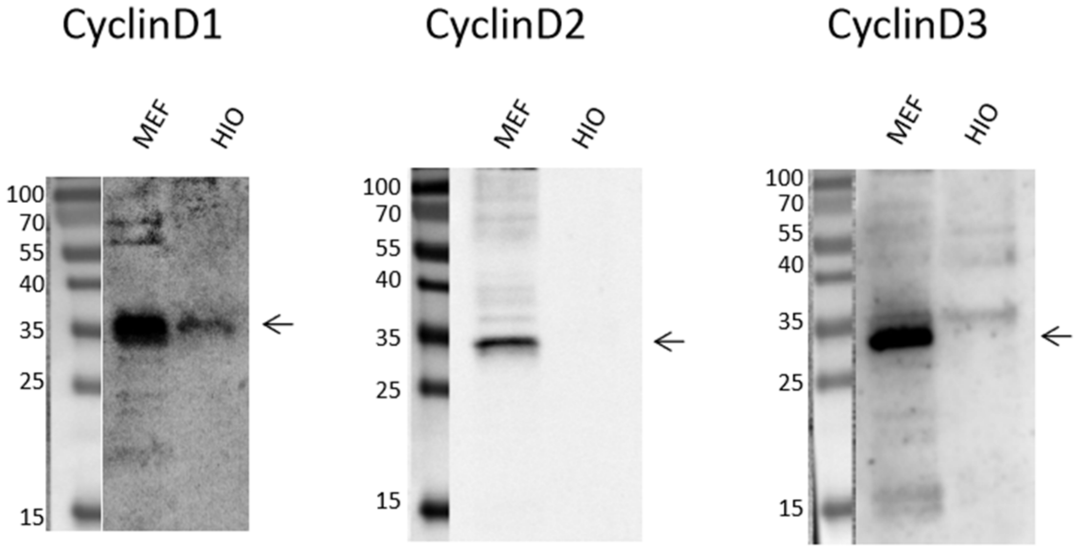

Fig. S1. The relative cellular concentrations of the indicated Cyclin D isoforms were determined in lysates from human intestinal organoids (HIOs) and Rac1$^{fl/fl}$ MEFs using Western blot analysis. Representative immunoblots are presented (n=3).

Fig. S2

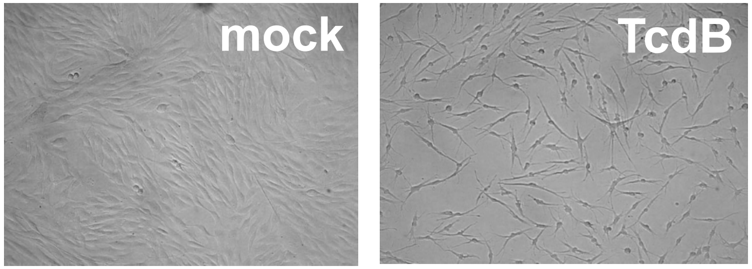

Fig. S2. The morphology of HCEC treated with 10 ng/ml TcdB for 24 h or left non-treated were analyzed using phase contrast microcopy. Representative morphologies were presented.

Fig. S3

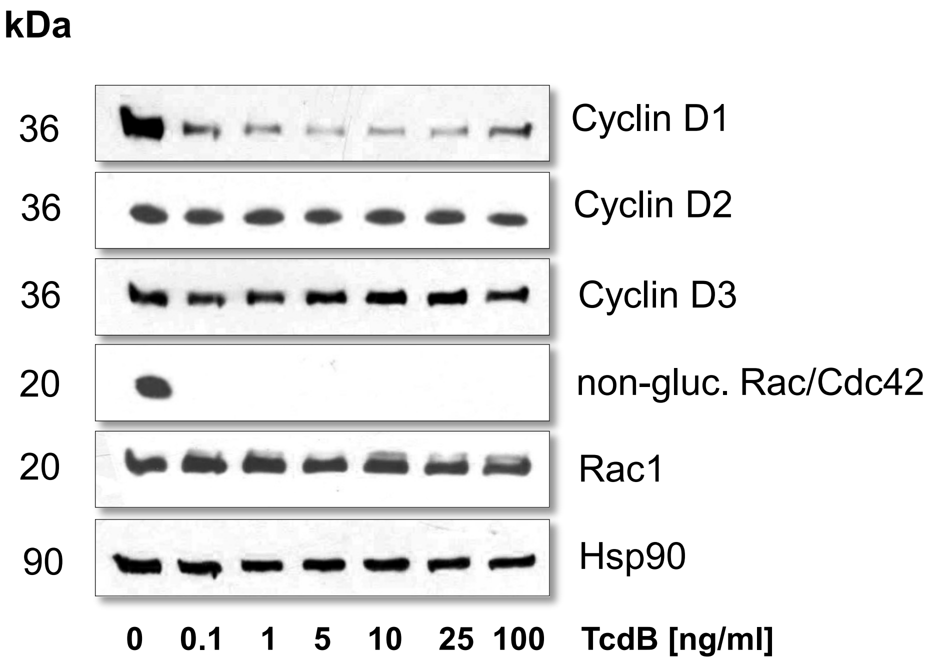

Fig. S3. HCEC were treated with the indicated concentrations of TcdB for 24 h. The relative cellular concentrations of the indicated proteins were determined using Western blot analysis. Representative Western blots are presented.

Fig. S4

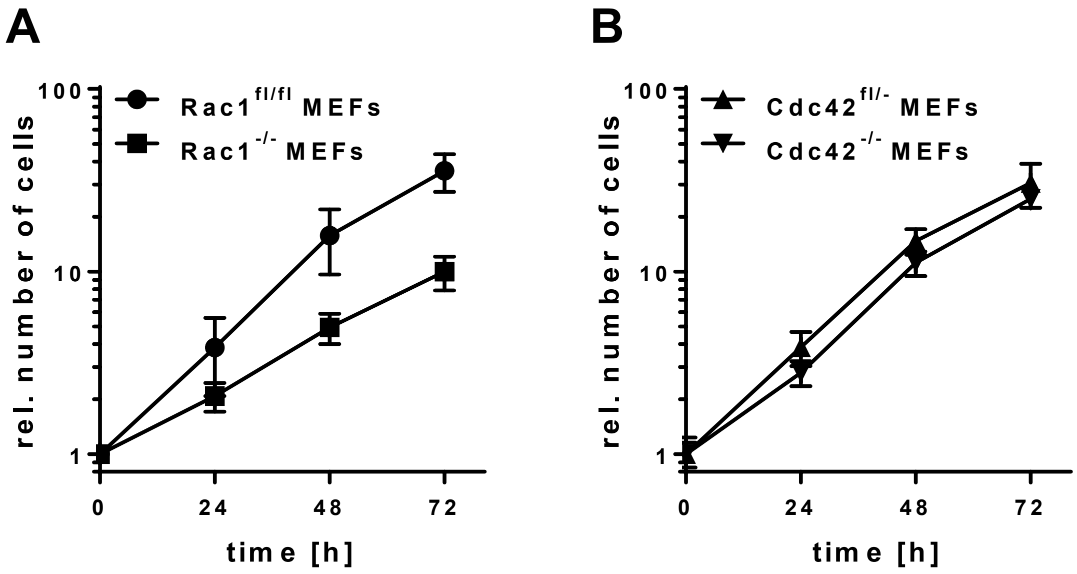

Fig. S4. The number of cells of proliferating $Rac1^{fl/fl}$ and $Rac1^{-/-}$ MEFs (A) and of proliferating $Cdc42^{fl/-}$ and $Cdc42^{-/-}$ MEFs (B) was determined by manual counting using a Neubauer chamber. The number of cells at time = 0 was set 1.0.

Fig. S5

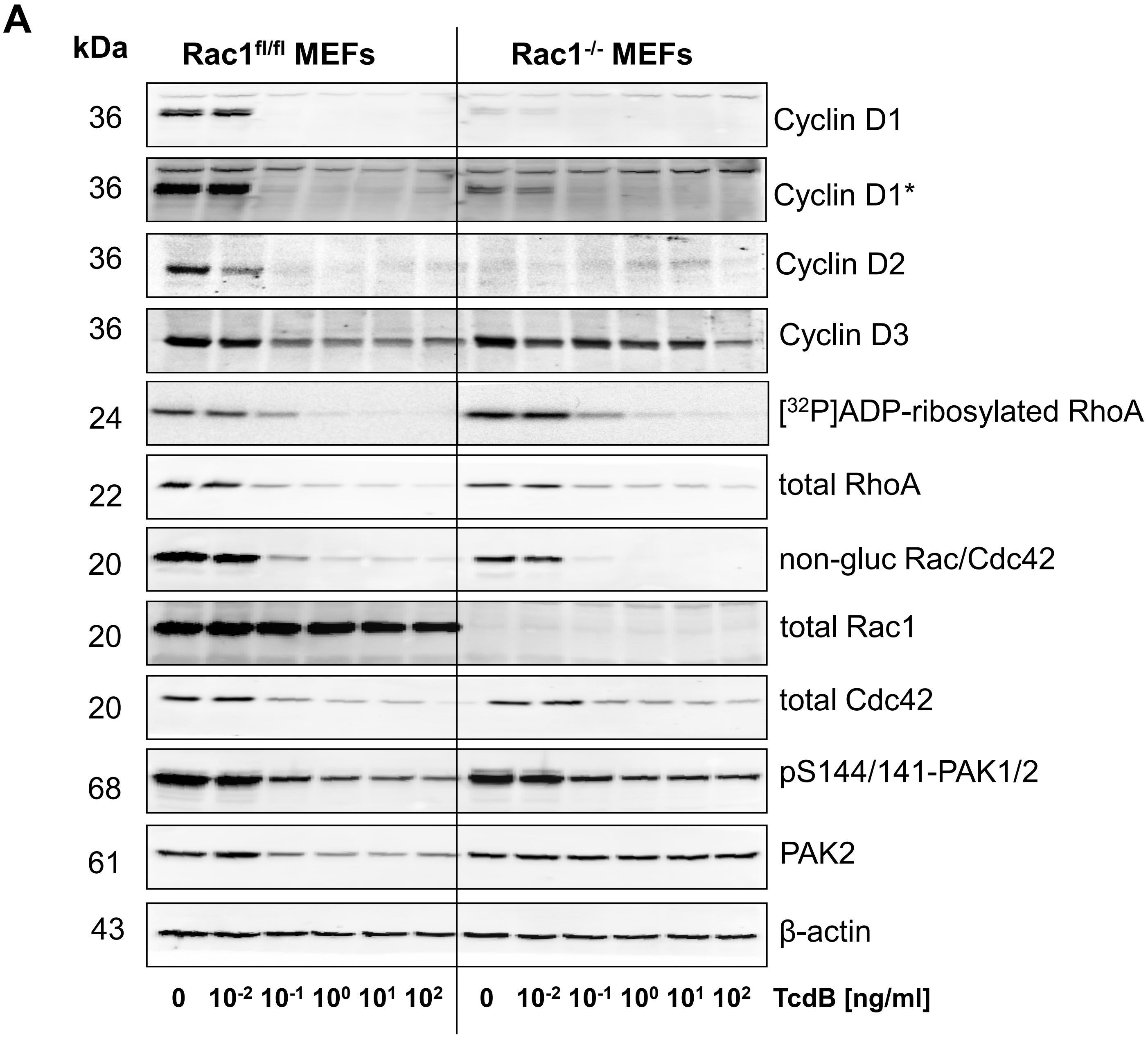
A
kDa
Rac1fl/fl MEFs
Rac1-/- MEFs
36
36
36
36
24
22
20
20
20
68
61
43
Cyclin D1
Cyclin D1*
Cyclin D2
Cyclin D3
[32P]ADP-ribosylated RhoA
total RhoA
non-gluc Rac/Cdc42
total Rac1
total Cdc42
pS144/141-PAK1/2
PAK2
β-actin
0 10-2 10-1 100 101 102
0 10-2 10-1 100 101 102
TcdB [ng/ml]

B

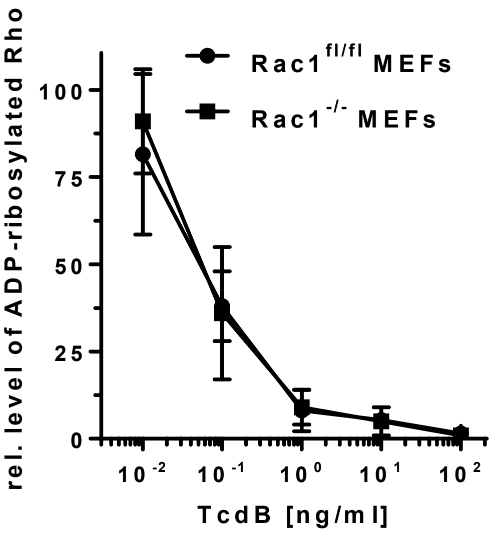

Fig. S5. Suppression of Cyclin D1 and Cyclin D2 in TcdB-treated fibroblasts. A. Proliferating Rac1$^{fl/fl}$ and Rac1$^{-/-}$ MEFs were treated with the indicated concentrations of TcdB for 24 h. The relative cellular concentrations of respective proteins in distinct cell lines were determined using Western blot analysis, as indicated. Representative Western blots out of three independent experiments are presented. The Cyclin D1 immunoblot is additionally provided in a version (*) with accentuated contrast to better visualize the low level of residual Cyclin D1 present in non-treated Rac1$^{-/-}$ MEFs. For the analysis of the relative concentrations of glucosylated RhoA/B/C, cells were then lysed and incubated with *C. botulinum* C3 exoenzyme in the presence of 1 µM [^{32}P]NAD for 30 minutes. A representative autoradiograph of the SDS-PAGE showing [^{32}P]ADP-ribosylated RhoA is presented. Decreasing signals of [^{32}P]ADP-ribosylated RhoA/B/C reflect increasing RhoA/B/C glucosylation. B. Signal intensities obtained from [^{32}P]ADP-ribosylated RhoA/B/C were quantified (n=3) and normalized to the signal of beta-actin. The concentration of [^{32}P]ADP-ribosylated RhoA/B/C from non-treated cells was set 100. Values are given as mean ± SD of three independent experiments.

Fig. S6

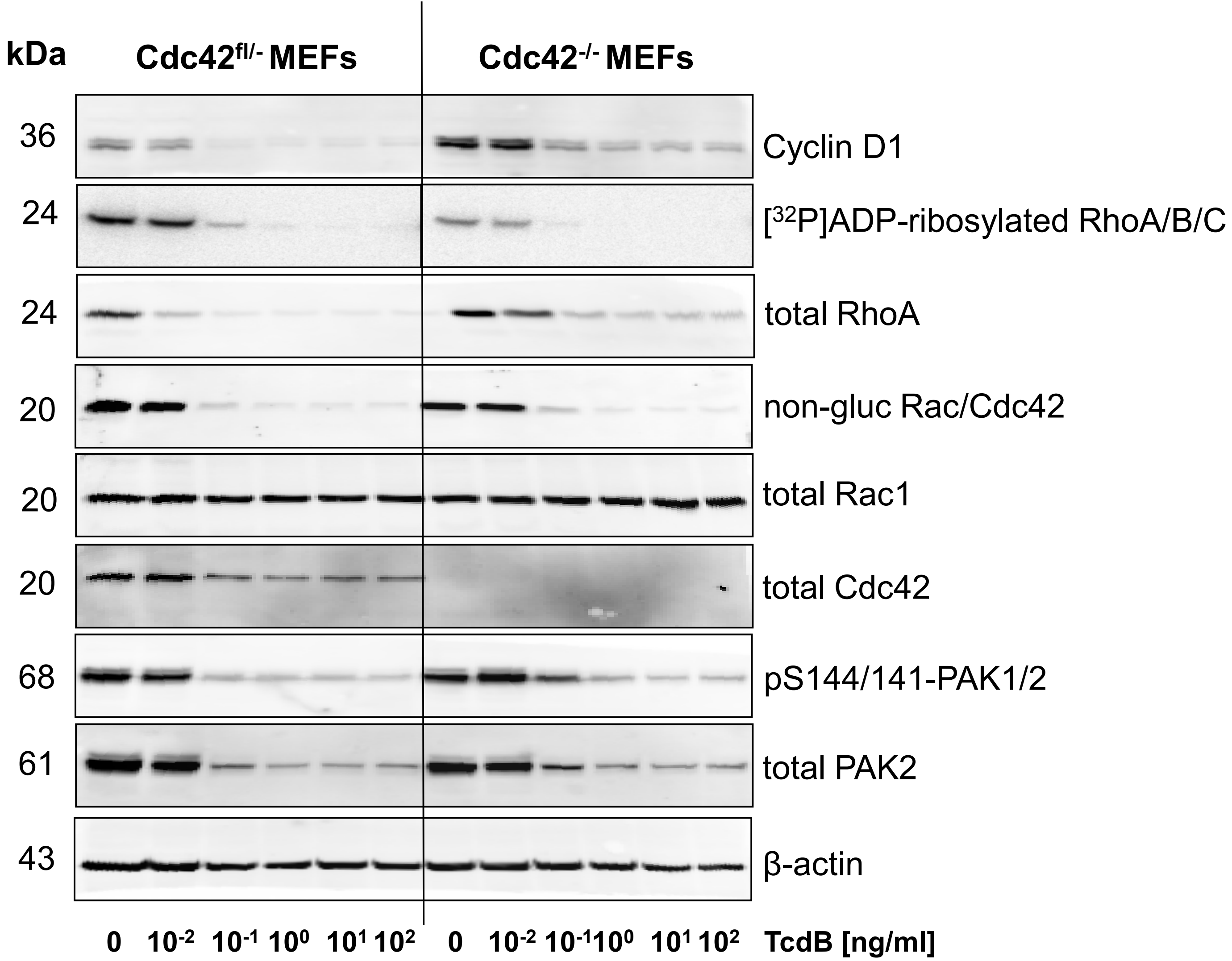

Fig. S6. Proliferating Cdc42$^{fl/-}$ and Cdc42$^{-/-}$ MEFs were treated with the indicated concentrations of TcdB for 24 h. The relative cellular concentrations of respective proteins in Cdc42$^{fl/-}$ and Cdc42$^{-/-}$ MEFs were determined by Western blot analysis. For analysis of the relative concentrations of glucosylated RhoA/B/C, cells were then lysed and incubated with *C. botulinum* C3 exoenzyme in the presence of 1 µM [^{32}P]NAD for 30 minutes. A representative autoradiograph of the SDS-PAGE showing [^{32}P]ADP-ribosylated RhoA/B/C is shown. Decreasing signals of [^{32}P]ADP-ribosylated RhoA/B/C and of non-glucosylated Rac/Cdc42 reflect increasing RhoA/B/C glucosylation and Rac/Cdc42 glucosylation, respectively.

Fig. S7

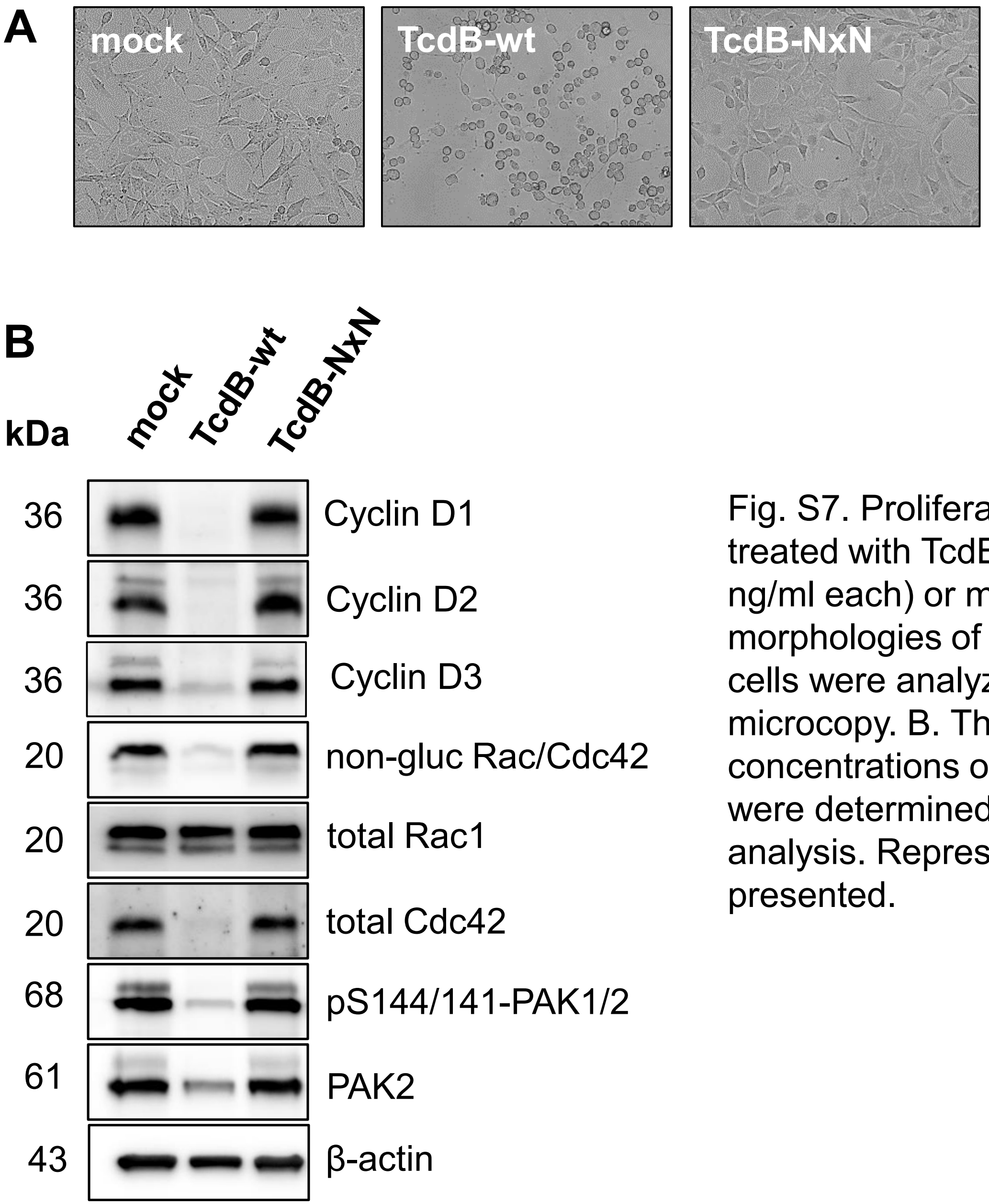

Fig. S7. Proliferating Rac1$^{fl/fl}$ MEFs were treated with TcdB-wt and TcdB-NxN (1 ng/ml each) or mock for 24 h. A. The morphologies of toxin- or mock-treated cells were analyzed using phase contrast microcopy. B. The relative cellular concentrations of the indicated proteins were determined using Western blot analysis. Representative Western blots are presented.

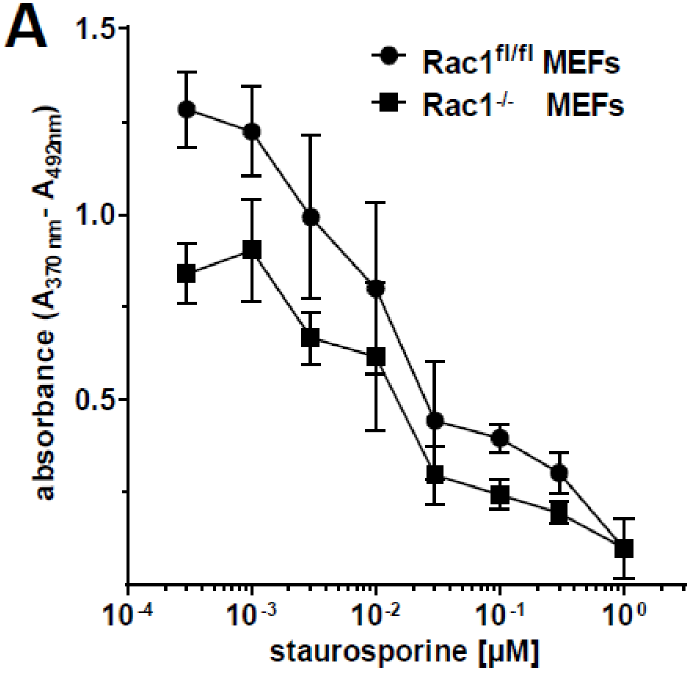

Fig. S8

Fig. S8. Effects of staurosporine in Rac1$^{fl/fl}$ and Rac1$^{-/-}$ MEFs. A. Proliferating Rac1$^{fl/fl}$ and Rac1$^{-/-}$ MEFs were labelled with BrdU (10 µM) and treated with the indicated concentrations of staurosporine (STS) for 24 h. DNA *de novo* synthesis was determined using a peroxidase-conjugated anti-BrdU antibody. B. Proliferating Rac1$^{fl/fl}$ and Rac1$^{-/-}$ MEFs were treated with the indicated concentrations of staurosporine (STS) for 24 h. The relative cellular concentrations of respective proteins in distinct cell lines were determined by Western blotting as indicated.

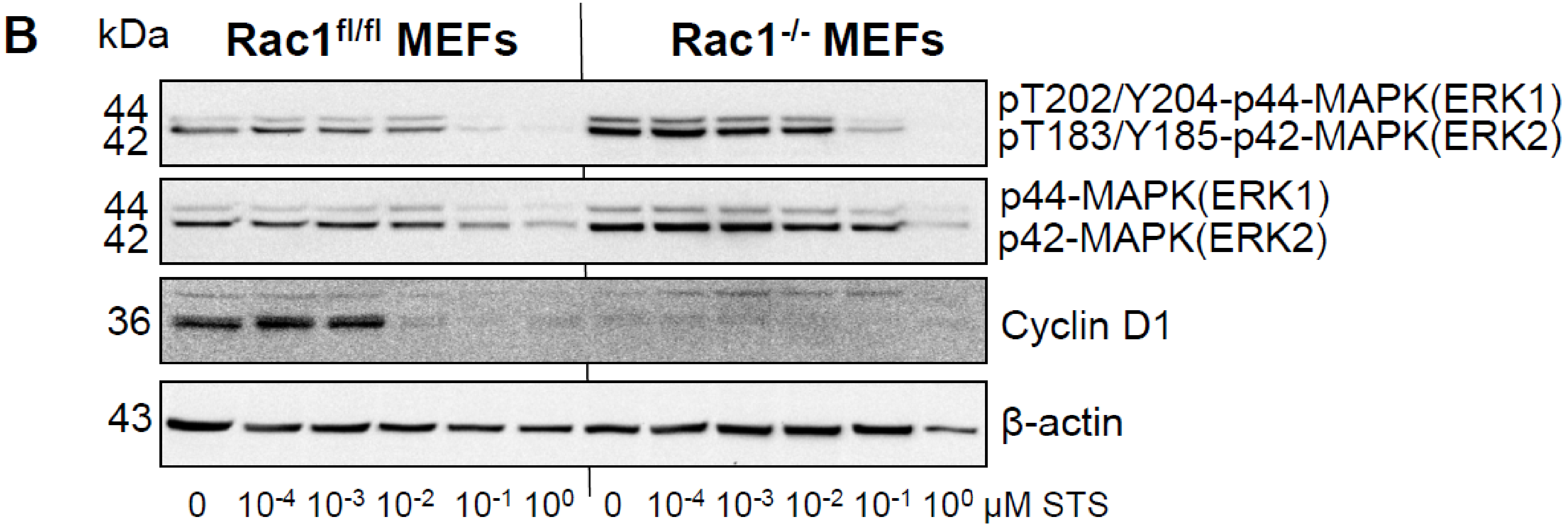

Supplement: Supplementary file 1 [file Data_Sheet_1.pdf]
